# Home Videos as a Cost-Effective Tool for the Diagnosis of Paroxysmal Events in Infants: Prospective Study

**DOI:** 10.2196/11229

**Published:** 2019-09-12

**Authors:** Lu-Lu Huang, Yang-Yang Wang, Li-Ying Liu, Hong-Ping Tang, Meng-Na Zhang, Shu-Fang Ma, Li-Ping Zou

**Affiliations:** 1 Chinese People's Liberation Army General Hospital Beijing China; 2 Chang-De First People’s Hospital Changde China

**Keywords:** paroxysmal events, infant, home videos, online consultation

## Abstract

**Background:**

The diagnosis of paroxysmal events in infants is often challenging. Reasons include the child’s inability to express discomfort and the inability to record video electroencephalography at home. The prevalence of mobile phones, which can record videos, may be beneficial to these patients. In China, this advantage may be even more significant given the vast population and the uneven distribution of medical resources.

**Objective:**

The aim of this study is to investigate the value of mobile phone videos in increasing the diagnostic accuracy and cost savings of paroxysmal events in infants.

**Methods:**

Clinical data, including descriptions and home videos of episodes, from 12 patients with paroxysmal events were collected. The investigation was conducted in six centers during pediatric academic conferences. All 452 practitioners present were asked to make their diagnoses by just the descriptions of the events, and then remake their diagnoses after watching the corresponding home videos of the episodes. The doctor’s information, including educational background, profession, working years, and working hospital level, was also recorded. The cost savings from accurate diagnoses were measured on the basis of using online consultation, which can also be done easily by mobile phone. All data were recorded in the form of questionnaires designed for this study.

**Results:**

We collected 452 questionnaires, 301 of which met the criteria (66.6%) and were analyzed. The mean correct diagnoses with and without videos was 8.4 (SD 1.7) of 12 and 7.5 (SD 1.7) of 12, respectively. For epileptic seizures, mobile phone videos increased the mean accurate diagnoses by 3.9%; for nonepileptic events, it was 11.5% and both were statistically different (*P*=.006 for epileptic events; *P*<.001 for nonepileptic events). Pediatric neurologists with longer working years had higher diagnostic accuracy; whereas, their working hospital level and educational background made no difference. For patients with paroxysmal events, at least US $673.90 per capita and US $128 million nationwide could be saved annually, which is 12.02% of the total cost for correct diagnosis.

**Conclusions:**

Home videos made on mobile phones are a cost-effective tool for the diagnosis of paroxysmal events in infants. They can facilitate the diagnosis of paroxysmal events in infants and thereby save costs. The best choice for infants with paroxysmal events on their initial visit is to record their events first and then show the video to a neurologist with longer working years through online consultation.

## Introduction

Paroxysmal events in infants are characterized by sudden, mostly short-term, involuntary movements involving various parts of their body [1-3]. Some are epileptic seizures, whereas some are nonepileptic seizures resulting from immaturity of the central nervous system or other pathological or nonepileptic mechanisms [4,5]. Approximately 20% to 40% of patients in epilepsy referral centers are diagnosed with paroxysmal nonepileptic events [6]. Bye et al [3] reported that paroxysmal nonepileptic events are diagnosed in 43% of children who underwent video electroencephalography (VEEG) monitoring. However, there are still difficulties in accurately diagnosing paroxysmal nonepileptic events in this population [7,8]. First, because infants are unable to express their complaints; seizure descriptions are mostly made by their caregivers, who have never been trained to identify seizure types. Thus, the descriptions may be inaccurate or incomplete [9]. Second, without video recordings it is more difficult to make a diagnosis [10].

Video EEG, the gold standard in the differential diagnosis of paroxysmal events [11,12], plays a central role in clinical work [10,13-15]. With VEEG, the diagnostic accuracy rate can reach up to 88.0% [16], whereas that of ambulatory EEG is only 67.5% [17]. However, paroxysmal events usually occur at home with no aura, so a VEEG test monitoring the paroxysmal events is unavailable in most cases. Worse still, many centers in China do not own VEEG test facilities, so some sporadic events may never be recorded. In such cases, clinical acumen based on the description of the events is the only assistance clinicians have to make their diagnoses [16].

In China, the contradiction between the surging number of children and the lack of pediatricians, and between the concentrated distribution of high-level hospitals (mostly in developed areas) and widespread distribution of patients (many in remote areas) makes it difficult for many patients to get a timely diagnosis and follow-up treatment. Except for Beijing and Shanghai, the supply-and-demand ratios of pediatricians are less than 0.80, and there is a need for another 191,981 to 198,287 pediatricians [18]. Another harsh reality is that family of patients from remote and less-developed areas have to spend a lot of money on traffic and accommodation fees. All these have greatly exacerbated the issue of “difficulties and high costs of getting medical services” in China [19].

With the increasing popularity of mobile phones worldwide, recording the paroxysmal events has become easier. With mobile phones, patients can choose an online consultation instead of visiting a traditional outpatient clinic, which will greatly decrease their costs. There have been many studies on the value of home video and its contribution to increasing diagnostic accuracy [20,21]. However, none focused on the value of home video for paroxysmal events in infants. Also, none provided patients with guidance on what doctors they should visit after recording the events.

This study aims to identify the value of home videos for the diagnosis of paroxysmal events and its potential use for online consultation and providing guidance about which doctor to choose when patients or their caregivers have recorded events and chosen online consultation.

## Methods

### Study Design

The study is a prospective study with three steps: investigating the value of the home videos for diagnosis on the patients’ first visit, the cost savings for these patients with videos when choosing online consultation, and the type of doctor they should choose.

The trial was conducted following the international rules of good clinical practice. The study was approved by the General Hospital of the People’s Liberation Army. Informed consent was obtained from each patient’s parents. The parents would describe the paroxysmal events to the senior epileptologist, who would edit the description to meet the criteria of the clinical medical record and include any relevant personal or family history. Therefore, each patient’s description information was complete and would accurately simulate the patient’s consultation process in the outpatient clinic.

### Video Selection

**Figure 1:**
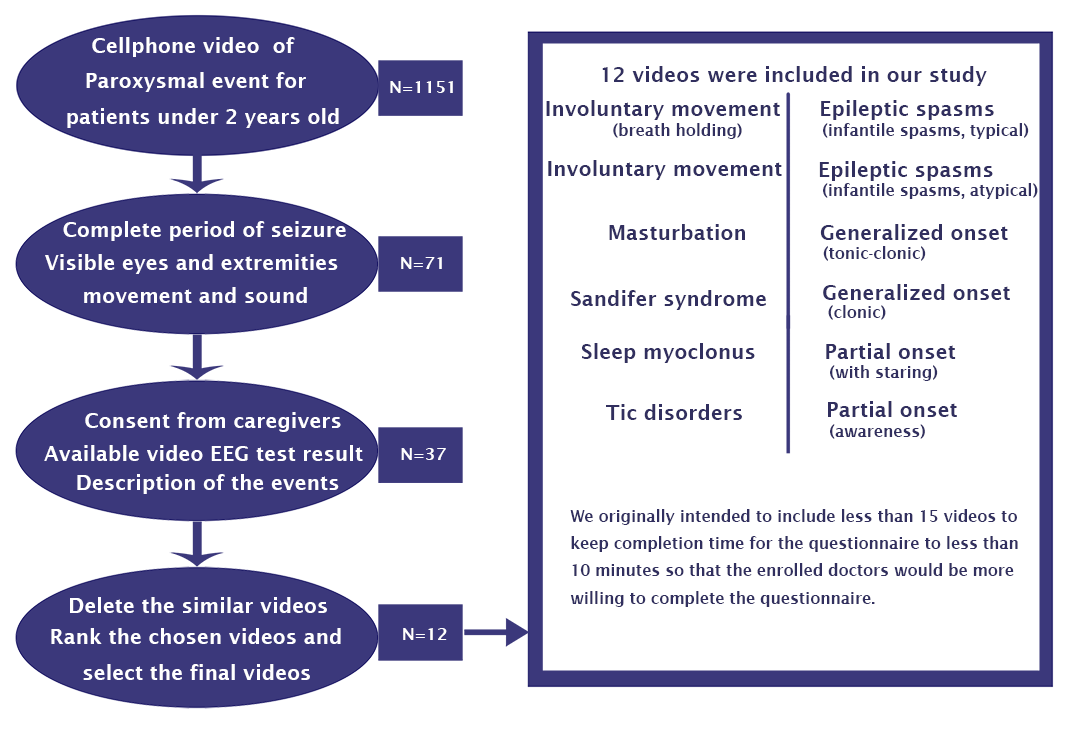
Flowchart for selecting qualified videos.

Twelve video recordings showing various paroxysmal events in infants were collected from the Chinese PLA General Hospital outpatient clinic from May 2015 to January 2016. The inclusion and exclusion criteria are listed in Textbox 1. Similar videos were removed until the shortest remained, but we ensured that each typical episode was presented. The flowchart for choosing the videos is presented in Figure 1. The VEEG reports of all 12 patients were also collected.

**Inclusion and exclusion criteria for the videos.**

**Inclusion criteria**

1. The resolution of the videos is high enough to ensure that the patient’s facial features are visible;
2. All possible body movements of the patient were recorded; and
3. The sound in the videos is clear, and whether there is excessive ventilation can be distinguished.

**Exclusion criteria**

1. No consent has been achieved from the patients’ caregivers; and
2. The video is longer than 1 minute (may affect the efficiency of public playback).

All corresponding descriptions, home videos, and VEEG reports were presented to two senior epileptologists blind to the study purpose, and they made diagnoses accordingly. Events were categorized as epileptic or nonepileptic: if epileptic, the specific seizure type was listed; if nonepileptic, a diagnosis to explain the paroxysmal events was given. When the diagnoses from the two epileptologists were not the same, a third epileptologist would review the data and provide the diagnoses. We did not encounter a situation in which all three reviewers could not achieve an agreement.

### Data Collection

**Figure 2:**
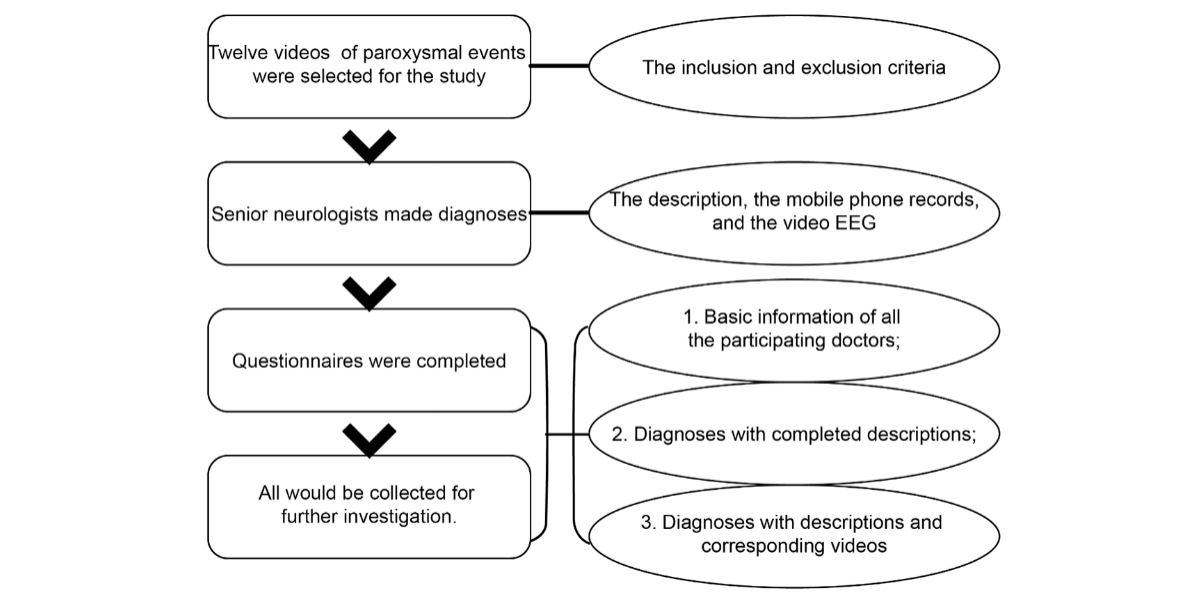
Flowchart of the study of the value of videos. EEG: electroencephalography.

We conducted our investigation in six centers during the pediatric academic conferences. The person playing the videos was unaware of the diagnosis. Figure 2 shows the three steps of the study. A questionnaire was designed to simulate the process of a clinic consultation. The first part of the questionnaire was the basic information of the doctors, including their educational background, profession (pediatrician or pediatric neurologist), working hospital levels (first/secondary/tertiary hospital), and working years. This was because, in clinical work, the doctor’s basic information is open to all patients visiting the outpatient clinic. The second part provided the doctors with the description of the episodes. This part simulated the process of collecting the medical record at the beginning of the patient’s visit. The description provided all the information that the patient would provide to the clinician when there was not a video. The doctors were required to make their diagnoses and fill in the questionnaire. It should be noted that we originally required the doctor to identify the specific type of epilepsy when they considered it an epileptic seizure, but when we simulated the process in our hospital, we found that the general pediatricians could not clearly identify the types of epilepsy. Therefore, we deleted this part and only required the doctors to identify the episodes as epileptic or nonepileptic. The third part of the questionnaire presented the same descriptions as the second part, but the difference was that in the process of actually collecting these data, the doctors would simultaneously see the corresponding videos before making their diagnoses. Before the investigation, the enrolled doctors were informed of the purpose of the study; all data were anonymous. Only the completed and identifiable questionnaires were eligible for our study.

### Data Analysis

**Figure 3:**
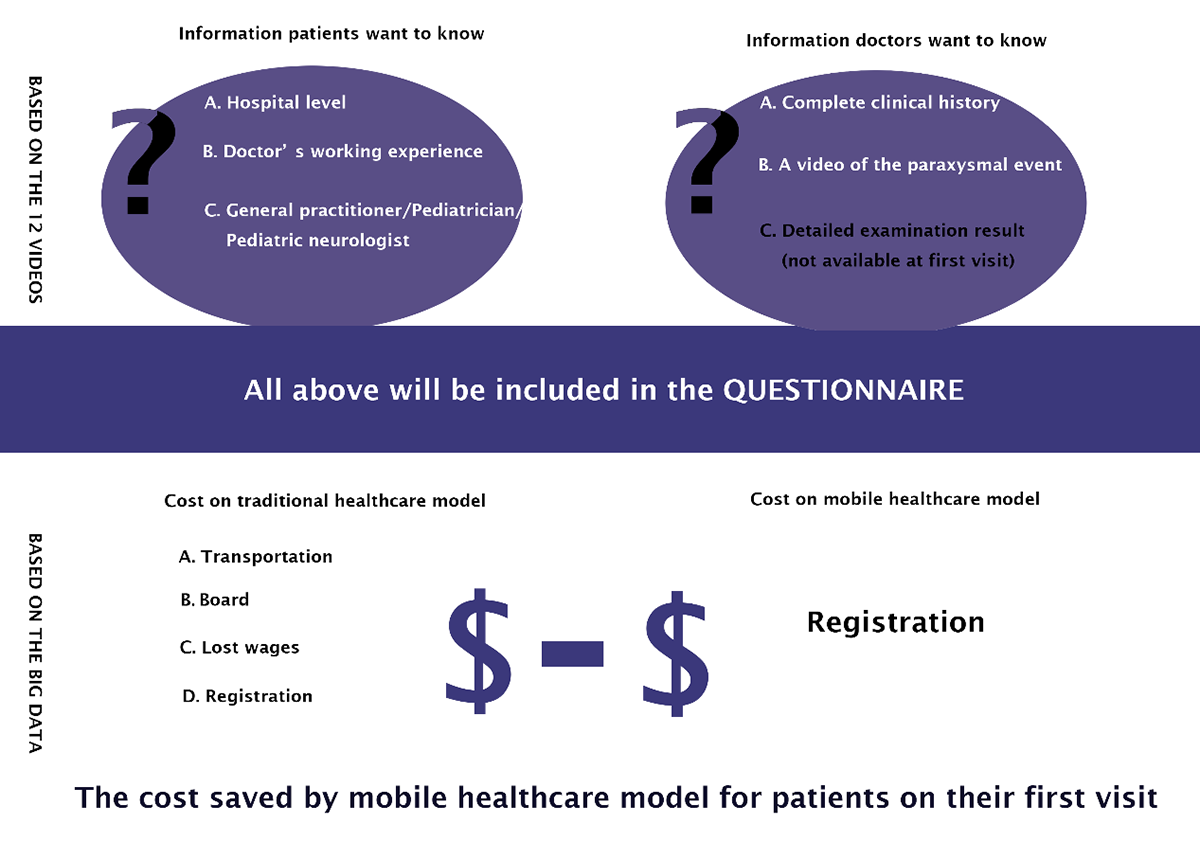
The simulation process and the value of videos on cost savings.

We calculated the cost savings with the help of home videos on the basis of the contribution videos made on correct diagnoses. In this part, we used the model of online consultation instead of the traditional outpatient visit because this is the increasingly popular choice in China with the popularity of mobile phones [22]. Also, we believed that online consultation is a more reasonable choice considering the difficulties and high costs of getting medical services in China, especially for patients from remote areas [23-25]. The cost savings by online consultation and for patients with or without videos were obtained from published articles or government work reports, including transportation fees, accommodation fees, loss of wages, and registration fees. Figure 3 illustrates this simulation process and how the cost savings were calculated.

All data were recorded in Epidata and exported to SPSS version 24 for statistical analysis. Calculations for the correct diagnosis percentage for each episode were made, and the difference was compared by chi-square test. For the impact of the doctors’ background on diagnosis, we gave a correct diagnosis a value of 1 and an incorrect diagnosis a value of 0, and then calculated each doctor’s score with and without the help of home videos. We then compared the influence of doctors’ profession, educational background, working hospital level, and working years on accurate diagnosis by multiple linear regression analysis. We further analyzed the scores of each physician on the diagnosis of epileptic seizures and nonepileptic seizures with or without videos to analyze the role of the doctor’s background information in both cases. The statistical tests presented are two-sided; a *P* value less than .05 was considered statically significant.

All patients, if they consult online for their first visit, can save the cost of transportation, accommodation, and loss of wages. Because there has not been any standard for the charge of online consultation in China, we equated the fee to the traditional outpatient registration fee, so it could not be saved. There are also no data on the number of pediatric patients with epilepsy; therefore, we first estimated the annual incidence and the total number of new epilepsy patients through related literature [12,26-32]. Then, we calculated the total number of new patients annually who presented with a paroxysmal event and were diagnosed as a nonepileptic event according to the ratio of the epileptic events to nonepileptic events in all paroxysmal events in children [33,34]. The proportion of infant patients was not available, so when calculating the total cost savings of bringing videos and choosing online consultation, we extended the age to 9 years and calculated the total cost savings for patients younger than 9 years nationwide. In addition, if the patients did not obtain accurate diagnoses on their first visit, they would have to pay additional charges on a later visit. We will never know how many visits these patients needed before accurate diagnosis; therefore, we assumed that they would all achieve an accurate diagnosis on their second visit so the cost savings would be the minimum. We then compared the cost savings of bringing videos with that of just a description to study the value of videos. The formulas are presented in Multimedia Appendix 1.

## Results

### Mobile Phone Videos and Diagnosis Accuracy

A total of 452 questionnaires were collected, 301 of which met the criteria (66.6%) and were included in the study. Table 1 shows the demographic characteristics of the whole study sample.

The mean number of correct diagnoses through descriptions only and descriptions with home videos was 7.5 (SD 1.7) of 12 and 8.4 (SD 1.7) of 12, respectively. Details of the percentages of correct diagnoses are shown in Table 2.

**Table 1:** Demographic characteristics of the whole study sample (N=301).

Demographic items		Study sample
Age (months), mean (SD)		16.0 (37.1)
**Gender, n (%)**		
	Male	79 (26.2)
	Female	222 (73.8)
**Hospital, n (%)**		
	Secondary level	93 (30.9)
	Tertiary level	208 (69.1)
**Duration of working, n (%)**		
	<10 years	149 (49.5)
	≥10 years	152 (50.5)
**Profession, n (%)**		
	Pediatrician	218 (72.4)
	Pediatric neurologist	83 (27.6)

**Table 2:** Classification of paroxysmal events and percentage of doctors who correctly identified the episodes (N=301).

Video		Seizure classification	Correct identification, n (%)		*P* value
			Description only	Description with video	
**Epileptic**						
	1	Partial	266 (88.4)	262 (87.0)	.78
	3	Spasm	233 (77.4)	261 (86.7)	.048
	5	Generalized	262 (87.0)	291 (96.7)	.04
	7	Partial	274 (91.0)	267 (88.7)	.62
	10	Generalized	260 (86.4)	262 (87.0)	.89
	11	Spasm	232 (77.1)	260 (87.0)	.048
**Nonepileptic**						
	2	Involuntary movements	95 (31.6)	178 (59.1)	<.001
	4	Sleep myoclonus	204 (67.8)	230 (76.4)	.07
	6	Tic disorders	152 (50.5)	198 (65.8)	.001
	8	Sandifer syndrome	110 (36.5)	86 (28.6)	.09
	9	Involuntary movements	85 (28.2)	168 (55.8)	<.001
	12	Masturbation	83 (27.6)	119 (39.5)	.01

For epileptic events, the mean percentage of correct diagnoses with only a description available was 84.9% (SD 5.9%), and with a home video it was 88.8% (SD 3.9%). The difference was statistically significant (*P*<.001). For nonepileptic events, the mean percentage of correct diagnoses with only a description available was 39.9% (SD 15.9%), and with a home video it was 51.4% (SD 17.5%). The difference was also statistically significant (*P*<.001).

### Cost Savings With Home Videos

The means of the transportation fees, accommodation fees, further examination fees, and loss of wages according to the published literature and governmental reports [26-31] were US $290.00 (SD $56.14), US $86.30 (SD $22.54), US $192.90 (SD $61.73), and US $104.70 (SD $90.02), respectively. The annual new pediatric patient (younger than 9 years) population is 197,945 in China. Online consultation could save US $1.28 million (8.22 million yuan) per year. For infants with paroxysmal events with videos on their first visit, at least 12.02% of the total cost can be saved.

### Which Doctor to Choose

In the analyses of doctor’s background information, we found that the number of working years was the key factor for a correct diagnosis. Whether or not videos were provided, the level of the doctor’s hospital and educational background were irrelevant to the correctness of the diagnosis. When only parents’ descriptions were available, profession was the only factor that affected the correctness of the diagnosis for epileptic seizures; for nonepileptic seizures, pediatric neurologists with relatively longer working years made more accurate diagnoses. When both descriptions and videos were available, profession and hospital level were the two factors that affected the correctness of the diagnosis for epileptic seizures; for nonepileptic seizures, working years and profession were related to the accuracy of the diagnosis. The statistical results are listed in Table 3.

**Table 3:** Variables of doctors’ backgrounds and the effect on correct diagnoses.

Variable			Standardized coefficients, beta (95% CI)	*P* value
**Description**				
	**Epileptic**			
		Education level	.042 (−.078, .162)	.49
		Hospital level	−.062 (−.186, .062)	.33
		Working years	−.096 (−.208, .017)	.09
		Profession	.125 (.005, .246)	.04
	**Nonepileptic**			
		Education level	.046 (−.073, .165)	.45
		Hospital level	−.056 (−.178, .066)	.37
		Working years	.194 (.083, .305)	.001
		Profession	.122 (.003, .241)	.04
	**Total**			
		Education level	.067 (−.050, .185)	.26
		Hospital level	−.088 (−.210, .033)	.15
		Working years	.118 (.008, .228)	.26
		Profession	.187 (.069, .304)	.002
**Video**				
	**Epileptic**			
		Education level	−.030 (−.149, .090)	.62
		Hospital level	−.152 (−.275, −.029)	.02
		Working years	.128 (.017, .239)	.02
		Profession	.113 (−.006, .233)	.06
	**Nonepileptic**			
		Education level	.053 (−.066, .173)	.38
		Hospital level	.008 (−.115, .131)	.90
		Working years	.144 (.012, .033)	.01
		Profession	.163 (.008, .043)	.008
	**Total**			
		Education level	.039 (−.077, .155)	.51
		Hospital level	−.065 (−.184, .055)	.29
		Working years	.204 (.096, .313)	<.001
		Profession	.216 (.100, .332)	<.001

## Discussion

It is better for patients to take videos of paroxysmal events for their first visit, whether they will be diagnosed with epilepsy or not. Videos contribute a lot for the differential diagnosis. In our study, the availability of home videos increased the mean correct diagnosis percentage by 3.9% for epileptic events and11.5% for nonepileptic events. Compared with descriptions alone, videos are better at reflecting all the information of paroxysmal events. In a previous study including 45 semiological signs that can be used to distinguish paroxysmal nonepileptic events from epileptic events, only six proved reliable, and eyewitness reports were unreliable [9]. Due to the lack of relevant knowledge, parents’ descriptions may exaggerate some clinical symptoms, which will influence doctors’ judgments. The videos recorded by the caregivers are relatively more objective and can avoid this situation.

If there are videos available, some patients will be diagnosed with nonepileptic seizures just by the video recording and there will be no need for them to perform more examinations. Although some will be diagnosed as epileptic seizures, the videos can also save their cost on examination because the video can aid interpretation of ambulatory EEG in approximately one-third of patients and they may no longer need to perform a VEEG test [35]. Thus, videos can help doctors make correct diagnoses easier and earlier, and the benefit of early diagnosis can sometimes be huge because it may improve the prognosis of epileptic infants [36]. For a paroxysmal event, the doctors may be able to identify it as a nonepileptic through video easily and another 11.5% of the nonepileptic patients and their families will then bear a lighter mental and financial burden from subsequent diagnoses and treatment.

When home videos are available, online consultation is a better choice for their first visit. Previous studies have shown that the incidence of epileptic misdiagnosis in online diagnosis and the treatment process was no different from that of the traditional outpatient process [37]. Moreover, online diagnosis and treatment have a considerable advantage in integrating medical resources and reducing patients’ costs [38-41]. For patients during their first visit, there have been no examination results available, especially EEG monitoring. They may spend a lot on transportation, accommodation, loss in wages, and registration only to find that doctors need more examination results and they need to record the events before making a final diagnosis. They have to return home or live near the hospital for several days before their appointment date for further examinations or to record the events. Many costs would have been saved if they had used the online consultation after recording the events. According to our study, online consultation could save US $673.90 per capita and US $1.28 million (8.22 million yuan) in total per year for these patients. If these patients recorded the paroxysmal events, at least 12.02% of the total cost for correct diagnosis could be saved.

Therefore, we recommend all parents of infant patients with paroxysmal events record the events and choose online consultation for their first visit. This method is more important for to nonepileptic patients because it not only reduces their economic burden, but also saves them from unnecessarily worrying about an epilepsy diagnosis.

After recording the events and choosing online consultation, the patients may question which doctor to visit. From our study, experienced doctors—especially experienced neurologists—rather than doctors in higher-level hospitals or with better-educated backgrounds, are the best candidates for patients on their first visit. For the first visit, the caregivers naturally think that the better-educated and experienced neurologists in the higher-level hospital are their first choice. However, the level of hospital and a doctor’s educational background were not important according to our study. Nagy et al [42] have reported that the diagnostic accuracy of first-year medical students was even lower than that of patients’ parents.

In this study, we included a special nonepileptic event, Sandifer syndrome, which is a type of gastroesophageal reflux associated with laryngospasm. In infants, it may be misdiagnosed as seizures because of the presence of limb posturing, abnormal eye movements, and even opisthotonus [43]. These events mostly occur in sleep and thus cause diagnostic difficulties [12]. In our study, instead of increasing the identification rates, the videos confused the clinicians, who thought it was an epileptic seizure. Therefore, VEEG is still necessary in some situations.

Our study excluded the videos with poor quality, but videos from patients’ caregivers may not be of high enough quality in real clinical work, and this will influence the diagnosis [44]. Although we emphasize the importance of recording videos, the quality of videos should also be considered.

There are some limitations of our study. First, although we tried to ensure that the descriptions were complete, we cannot fully equate the descriptions to a conventional outpatient visit. Second, the cost savings from our study are just the minimum because we will never know, for specific patients, how many visits they may need before a correct diagnosis.

Home videos made on mobile phones are a cost-effective tool for the diagnosis of paroxysmal events in infants. They can facilitate correct diagnosis and thereby save their cost. Therefore, the best choice for infants with paroxysmal events on their initial visit is to record their events first and then show the video to a neurologist with longer working years through online consultation.

## Appendix

Multimedia Appendix 1 Formulas for cost savings.

## Abbreviations

EEG: electroencephalography
VEEG: video electroencephalography
