# Supplementary material for: Home Videos as a Cost-Effective Tool for the Diagnosis of Paroxysmal Events in Infants: Prospective Study
Source: JMIR Mhealth Uhealth. 2019 Sep 12;7(9):e11229. doi: 10.2196/11229 (PMC6746063; doi:10.2196/11229)
Supplement: Multimedia Appendix 1 [file mhealth_v7i9e11229_app1.pdf]

The cost savings when patients choose online consultation for their first visit:

$(\sum_{n=1}^{n=6} p_i N_i) \cdot P_c \cdot (\Delta S_{ep} + \Delta S_{nonep} \cdot R_{nonep})$

The additional cost of traditional outpatient consultation compared with online consultation when videos are not available:

$S_1 = Ni \cdot (\Delta S_{ep1} \cdot P_{d\text{-}ep} + \Delta S_{nonep1} \cdot R_{nonep} \cdot P_{d\text{-}nep} + \Delta S_{ep2} \cdot (1 - P_{d\text{-}ep}) + \Delta S_{nonep2} \cdot R_{nonep} \cdot (1 - P_{d\text{-}nep}))$

The additional cost of traditional outpatient consultation compared with online consultation when videos are available:

$S_2 = Ni \cdot (\Delta S_{ep1} \cdot P_{v\text{-}ep} + \Delta S_{nonep1} \cdot R_{nonep} \cdot P_{v\text{-}nep} + \Delta S_{ep2} \cdot (1 - P_{v\text{-}ep}) + \Delta S_{nonep2} \cdot R_{nonep} \cdot (1 - P_{v\text{-}nep}))$

The cost savings of bringing videos and choosing online consultation:

$\frac{S1 - S2}{S1} \times 100\%$

In the four formulas, variable *n* represents the different areas (n1=Northern China; n2=Northwest China; n3=Southwest China; n4=Central China; n5=Eastern China; n6=Northeast China). Variable *p* represents the incidence of epilepsy in the region and *N* represents the total population in the region. $P_c$ represents the proportion of pediatric patients. $\Delta S_{ep}$ represents the cost savings for epileptic patients, and $\Delta S_{nonep}$ represents the cost savings for nonepileptic patients. $R_{nonep}$ represents the ratio of nonepileptic

seizures versus epileptic seizures in paroxysmal events. *Ni* presents the population of epileptic infants nationwide. $\Delta S_{ep1}$ represents the total cost for epileptic patients on their first visit; $\Delta S_{ep2}$ represents the number on their second visit. $\Delta S_{nonep1}$ represents the total cost for nonepileptic patients on their first visit; $\Delta S_{nonep2}$ represents the number on their second visit. $P_{d\text{-}ep}$ represents the percentage of correct diagnoses for epileptic patients without home videos of paroxysmal events on their first visit; $P_{d\text{-}nep}$ represents the percentage for nonepileptic patients. $P_{v\text{-}ep}$ represents the percentage of accurate diagnoses for epileptic patients when home videos are available; $P_{v\text{-}nep}$ represents the percentage for nonepileptic patients.
